# P-138. Retrospective Review of Multi-modality Imaging Utilization in the Diagnosis, Management, and Follow-up of Patients with Infective Endocarditis at a Military Treatment Facility

**DOI:** 10.1093/ofid/ofaf695.365

**Published:** 2026-01-11

**Authors:** Riley Pickett, John Kiley, Mary B Ford

**Affiliations:** Brooke Army Medical Center, Fort Sam Houston, TX; BAMC, San Antonio, Texas; Brooke Army Medical Center, Fort Sam Houston, TX

## Abstract

**Background:**

Infective endocarditis (IE) often presents with non-specific symptoms, requiring high clinical suspicion for diagnosis. While echocardiography is critical to diagnose IE, the necessity and utility of follow-up imaging is unclear. American Heart Association (AHA) IE guidelines recommend end of therapy (EOT) imaging to establish a new baseline. This study describes the epidemiology, diagnosis, and EOT imaging of patients with IE at Brooke Army Medical Center (BAMC).

**Table 1:**
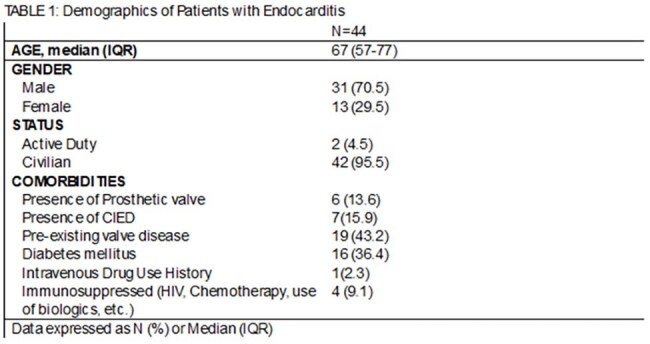
Demographics of Patients with Endocarditis

**Table 2:**
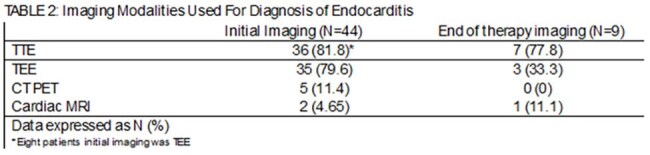
Imaging Modalities Used For Diagnosis of Endocarditis

**Methods:**

Patients >18 years of age admitted to BAMC from 1 Jan 2022 to 31 Oct 2024 were identified for inclusion by ICD codes for “Endocarditis” or “Infective Endocarditis”. Corresponding electronic health records were reviewed for epidemiologic, microbiologic, clinical, and imaging data. Patients with no available follow-up data were excluded.

**Table 3:**
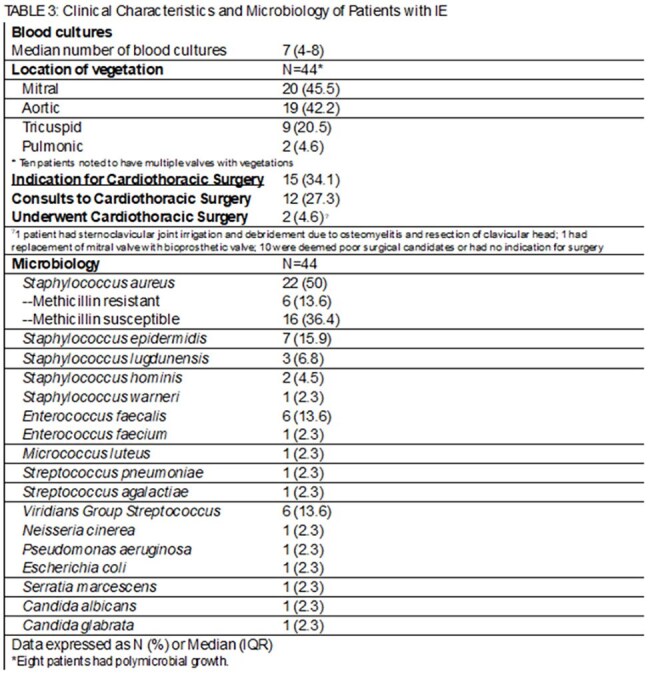
Clinical Characteristics and Microbiology of Patients with IE

**Results:**

Forty-four patients were included, majority male (70.5%) with a median age of 67. Thirteen (29.5%) had prosthetic material (valve or cardiac implantable electronic device [CIED]), only 1 reported intravenous drug use. Twenty-two patients (50%) had infections with *Staphylococcus aureus*, the majority were methicillin susceptible; 8 patients had polymicrobial infections. TTE was the initial imaging study for 36 (81.8%) patients, with TEE for the other 8. Twenty-eight patients (63.6%) required multiple imaging studies to confirm IE. Median duration of therapy was 42 days, and 12 patients died before EOT (27.3%). Nine of 32 patients alive at EOT had imaging (28.1%); 7 of these were for ongoing symptoms including fever and concern for septic emboli, with only 3 of those demonstrating new findings. Only 2 TTEs were obtained to establish a new baseline. At follow-up, 0 patients without EOT imaging had complications related to IE.

**Conclusion:**

In this study, *S. aureus* was the most common pathogen, most patients required multiple imaging studies to confirm the diagnosis, and less than a third of patients eligible for imaging had this completed at EOT. Additional studies are needed to guide recommendation on EOT imaging in asymptomatic patients.

**Disclosures:**

All Authors: No reported disclosures

